# Association of D-limonene and nisin: the action on vegetative cells and spores of *Alicyclobacillus* spp. in processed orange juice

**DOI:** 10.1007/s42770-026-02024-5

**Published:** 2026-07-24

**Authors:** Jean Lopes da Silva, Emilly Brito Ferreira, Laines Cassiano Sumera, Maria Fernanda Giugiolli, Evandro Bona, Danielle Lazarin Bidóia, Fabrícia Gimenes, Jane Martha Graton Mikcha, Beatriz Cervejeira Bolanho Barros, Benício Alves de Abreu Filho

**Affiliations:** 1 Graduate Program in Food Science, State University of Maringá, Av. Colombo, Maringá, 5790, 87020-900 Paraná Brazil; 2 Graduate Program in Sustainability, State University of Maringá, Av. Ângelo Moreira da Fonseca, Umuarama, 1800, 87506-370 Paraná Brazil; 3 Department of Technology, State University of Maringá, Umuarama Campus. Av. Ângelo Moreira da Fonseca, No. 1800, Parque Daniele, Umuarama, 87506370 PR Brazil; 4 Graduate Program in Food Technology (PPGTA), Federal Technological University of Paraná (UTFPR), Campo Mourão, Paraná Brazil; 5 Department of Clinical Analysis and Biomedicine, State University of Maringá, Av. Colombo, Maringá, 5790, 87020-900 Paraná Brazil; 6 Department of Basic Health Sciences, State University of Maringá, Av. Colombo, Paraná, 5790, 87020-900 Maringá Brazil

**Keywords:** *Alicyclobacillus* acidoterrestris, Antimicrobials, Essential oils, Limonene, Natural preservatives, Synergism

## Abstract

Contamination by *Alicyclobacillus* spp. is a challenge in the juice and acidic beverage industry, since it can cause spoilage and, consequently, economic losses. Natural preservatives are interesting strategies as an alternative search for microbial growth control in food, in particular the use of D-limonene (DL) and nisin (Nis). This study aimed to evaluate the antibacterial activity of the DL combination and Nis against five strains of *Alicyclobacillus* spp. *A. acidoterrestris* (CBMAI 0244^T^); *A. herbarius* (CBMAI 0246^T^); *A. acidiphilus* (CBMAI 0247^T^); *A. hesperidum* (CBMAI 0298^T^) *and A. sendaiensis* (KCTC 3843^T^). The interaction among the compounds was initially determined in culture medium by the checkerboard method, and the efficacy was evaluated by disk diffusion tests, Minimum Inhibitory Concentration (MIC), and Minimum Bactericidal Concentration (MBC). Subsequently, the combination was evaluated in orange juice to determine the Minimum Sporicidal Concentration (MSC). The checkerboard results against *A. acidoterrestris* revealed synergism, with a Fractional Inhibitory Concentration Index (FICI) of 0.07. The combination (DL + Nis) demonstrated larger inhibition halos (24–26 mm) compared to DL (10–11 mm) and Nis (20–24 mm) alone. The MIC values of the combination were reduced, ranging from 0.12 to 3.90 µg/mL which means it was bactericidal for all strains. In reconstituted orange juice, the combination was effective, with a MSC of 0.48 µg/mL against *A. acidoterrestris* spores. Electron microscopy revealed significant morphological damage and signs of cell lysis in treatments with the combination, suggesting a loss of membrane integrity consistent with the observed synergistic effect. Additionally, DL showed antioxidant activity of 0.23 µmol Trolox/100 g (ABTS), 91.67 µmol Trolox/100 g (FRAP), and electronic nose (E-nose) analysis demonstrated that the combination did not significantly alter the volatile profile of the juice. Therefore, the combination of D-limonene and nisin represents a natural alternative with potential for the control of *Alicyclobacillus* spp. in commercial orange juice.

**Supplementary Information:**

The online version contains supplementary material available at 10.1007/s42770-026-02024-5.

## Introduction

Microbial spoilage is one of the main causes of economic losses in the food industry, directly impacting the sensory quality, safety, and shelf life of products [1]. It is estimated that 30% of food produced globally is lost due to the action of microorganisms, which represents significant challenges for the production sector and the environment [2]. In the fruit juice segment, Brazil stands out as the world’s largest producer and exporter of concentrated orange juice. In the 2023/24 harvest, the country exported approximately 611,600 tons of Frozen Concentrated Orange Juice (FCOJ equivalent to 66°Brix), generating revenue of around US$1.66 billion. These figures reinforce the high economic relevance of maintaining product quality [3].

In this scenario, the *Alicyclobacillus* spp. emerges as one of the main spoilage microorganisms, being a recurring contaminant in orange, apple, tomato juices and teas [4]. This genus comprises Gram-positive, spore-forming, thermophilic, and acidophilic bacteria that are notorious for their ability to survive pasteurization processes and proliferate in the acidic environment of beverages [5]. Their proliferation results in the production of guaiacol and other volatile compounds, responsible for imparting atypical “off-flavors” and odors, making the product unfit for consumption and causing substantial losses to the industry [1].

Microbial control in the beverage industry has traditionally relied on the use of synthetic preservatives, such as benzoates and sorbates. However, with the growing consumer demand for “clean label” products, coupled with concerns about the potential adverse effects of chemical additives, has driven the search for natural and effective alternatives [6].

In this context, a variety of natural compounds have been explored, including plant extracts [7], fermentation-derived products such as kefir and kombucha [8, 9], and encapsulated enzymes [10].

D-limonene (4-isopropenyl-1-methylcyclohexene), a monoterpene present in more than 300 plant species, is the main constituent of citrus fruit essential oils. In addition to its acknowledged antimicrobial activity, it is classified as safe for food use (GRAS – Generally Recognized as Safe), which makes it a promising candidate for the industry [11].

Nisin, in turn, is a bacteriocin produced by *Lactococcus lactis* and is widely used as a food biopreservative due to its strong activity against Gram-positive bacteria. It is recognized as (GRAS) by the U.S. Food and Drug Administration (FDA), according to 21 CFR § 184.1538 which authorizes its use in foods under specified conditions [12]. Beyond its regulatory acceptance, nisin has been safely applied in the food industry for more than 50 years and is currently approved in over 50 countries, reflecting its long-standing record of efficacy and safety, nisin exhibits potent activity against several Gram-positive microorganisms, including *Alicyclobacillus* spp., but its antimicrobial spectrum is generally limited to this group, making it less effective against Gram-negative bacteria, yeasts, and molds [13, 14].

Therefore, the combination of natural compounds is a promising strategy to overcome individual limitations to obtain a synergistic effect, as already demonstrated for nisin with other essential oils [15, 16] The hypothesis is that D-limonene may destabilize the cell membrane of *Alicyclobacillus*, thus facilitating the action of nisin and enhancing its effect [17].

Therefore, this study aimed to evaluate the synergistic potential of the D-limonene and nisin combination both classified as Generally Recognized as Safe (GRAS) against different *Alicyclobacillus* strains. This association was investigated as a natural biopreservation strategy for orange juice, leveraging D-limonene’s relative sensory neutrality and antioxidant capacity to maintain product quality without the regulatory hurdles of synthetic additives.

## Materials and methods

### Microbial strains and culture conditions

The strains of from *Alicyclobacillus* spp. are from the Brazilian Collection of Environmental and Industrial Microorganisms (CBMAI), located at CPQBA/UNICAMP (São Paulo, Brazil). The species used were *Alicyclobacillus* spp. *A. acidoterrestris* (CBMAI 0244^T^); *A. herbarius* (CBMAI 0246^T^); *A. acidiphilus* (CBMAI 0247^T^); *A. hesperidum* (CBMAI 0298^T^) *and A. sendaiensis* (KCTC 3843^T^). The strains were stored in the Water Environment and Food Microbiology Laboratory of the State University of Maringá (UEM), in Maringá, Paraná. The stocks were maintained at − 20 °C in *Bacillus acidoterrestris* (BAT) medium supplemented with 30% (v/v) glycerol. For activation, the cultures were inoculated into 3 mL of BAT medium (pH 4.0), acidified with 0.1 M hydrochloric acid Sigma-Aldrich, and incubated at 45 °C for 24 h, according to Deinhard et al. [18].

### Preparation of the spore suspension

Vegetative cells of *A. acidoterrestris* were cultured in tubes containing BAT broth at pH 4.0 and 45 °C for 120 h. This incubation period was determined in preliminary tests to ensure a high yield of mature spores while maintaining a stable concentration of approximately ~ 10^^6^ spores/mL. The 80% sporulation threshold, verified by microscopy using the spore staining technique, was selected to provide a representative population of resistant spores for the subsequent antimicrobial assays. After incubation, the suspension was centrifuged (9.500 × g, 3 min), washed three times with sterile distilled water to remove residual nutrients and vegetative cell debris, and stored at 4 °C for a maximum of 48 h until use to ensure consistent viability and resistance. To verify the spore concentration and eliminate any remaining vegetative cells, the suspension was subjected to a water bath at 80 °C for 10 min. Following this heat shock, serial dilutions in 0.85% saline solution were plated on BAT agar and incubated at 45 °C for 48–72 h to allow for the full development of colonies from germinated spores, as slower-growing *Alicyclobacillus* spp. may require longer incubation times [19].

### Preparation of D-limonene solution

The D-limonene essential oil (~ 95% purity), extracted from orange peel, was provided by the Louis Dreyfus Commodities (LDC) (Paranavaí, PR, Brazil). The oil was stored in an amber bottle under refrigeration (4 °C) until use. For the tests, a stock solution was prepared at a concentration of 2000 µg/mL (v/v), pH 6.0 by dissolving it in 0.85% (w/v) saline solution and containing 0.1% (v/v) Tween 80 (Synth, Diadema, Brazil) as an emulsifying agent, used to improve the dispersion of the hydrophobic essential oil in the aqueous solution.

### Nisin solution

The commercial nisin from *Lactococcus lactis*, 2.5% (Sigma-Aldrich, Cat. No. N5764, St. Louis, MO, USA), was used. A stock solution was prepared at the time of use by dissolving 2000 µg of nisin in 1 mL of 0.02 M hydrochloric acid (HCl), pH 2.5 (Synth, Diadema, Brazil). The resulting solution, with a final concentration of 2000 µg/mL, was sterilized by filtration through a 0.22 μm acetate membrane.

### Disk diffusion susceptibility test

The sensitivity of the five *Alicyclobacillus* strains to the treatments was evaluated using the agar diffusion technique, based on the M07-A11guidelines of the Clinical and Laboratory Standards Institute [20]. The inoculum was prepared by suspending isolated colonies of each strain in saline solution (0.85%) until reaching a turbidity equivalent to the 0.5 standard on the McFarland scale (~ 1.5 × 10⁸ CFU/mL). With the use of a sterile swab, the suspension was uniformly distributed onto Petri dishes containing BAT agar. Sterile paper discs (6 mm in diameter) were placed on the inoculated agar, each impregnated with 20 µL of the test solutions (corresponding to a load of 20 µg/disc): D-limonene, nisin, or the combination of both. The negative control consisted of discs impregnated with the diluents used (0.85% saline and/or 1% Tween 80), ensuring they had no inhibitory effect. The positive control consisted of BAT agar inoculated with the bacterial suspension without the addition of antimicrobials, confirming strain viability and standardized growth, were incubated at 45 °C for 24 h. After incubation, antibacterial activity was evaluated by measuring the inhibition halos around the discs, with results expressed in millimeters (mm). Although 24 h was the standardized time for the final reading, all plates were monitored up to 72 h to confirm the stability of the inhibition halos.

### Minimum inhibitory concentration (MIC) and minimum bactericidal concentration (MBC)

MIC and MBC of the *Alicyclobacillus* spp. strains were determined according to protocol M07-A11 [20]. For the assay, a bacterial inoculum of each strain was prepared by adjusting the suspension in 0.85% saline solution to the turbidity standard of 0.5 on the McFarland scale. This suspension was then diluted in saline to achieve approximately 5 × 10⁵ CFU/mL. In 96-well microplates (TPP^®^, Switzerland), The serial dilutions ranging from 1000 to 0.0001 µg/mL were applied to both D-limonene and nisin, as well as to their combination (maintaining the same concentration range for each agent within the mixture), followed by the inoculation of 5 µL of the diluted bacterial suspension into each well. The assay included growth controls (inoculum without treatment) and sterility controls (medium without inoculum), using BAT medium. The plates were incubated at 45 °C for 24 h. Although 24 h is the standardized reading time, all microplates were monitored for up to 72 h to ensure that no late growth occurred for slow-growing strains. The MIC was defined as the lowest concentration capable of inhibiting visible growth. To determine the MBC, 10 µL aliquots from the unturbid wells were plated on BAT agar. After a further 24 h incubation, the MBC was considered to be the lowest concentration that prevented colony growth.

### Determination of the minimum sporicidal concentration (MSC)

The *A. acidoterrestris* spore suspension was prepared as described in Sect. 2.2. The MSC assay was performed in 96-well microplates, following protocol M07-A11 [20], with modifications. The wells contained serial dilutions of the treatments (D-limonene, nisin, and their combination) in BAT broth, with concentrations ranging from 0.49 to 1000 µg/mL. For the D‑limonene treatments, the medium was supplemented with 1% Tween 80 to improve the dispersion of the hydrophobic compound and to minimize spore aggregation, ensuring a homogeneous distribution of spores in the wells. Each well received 5 µL of the spore suspension, resulting in a final concentration of approximately 10⁴ spores/mL. The plates were incubated at 45 °C for 24 h. To account for the potentially slower growth of certain strains, growth was also monitored for up to 72 h; however, no changes in the results were observed after the initial 24 h reading. After this period, a thermal shock in a water bath (80 °C for 10 min) was applied to activate spore germination. Next, 10 µL from each well were seeded onto BAT agar and incubated at 45 °C for an additional 24 h. The MIC was defined as the lowest concentration of the treatment capable of inhibiting colony growth [21].

### Checkerboard method

To evaluate the antibacterial interaction between D-limonene and nisin, the methodology described by Eliopoulos and Moellering et al. [22] was used, based on the microdilution assay in 96-well plates for determining the Fractional Inhibitory Concentration Index (FICI). The combinations were tested at different concentrations, defined as the individual MIC values of each compound. After preparation, 5 µL of an *A. acidoterrestris* suspension (5 × 10⁴ CFU/mL) were added to each well. The plates were incubated at 45 °C for 24 h. The FICI indices were calculated as: FICI = FICI _A₁_ + FICI _B₂_; where FICI _A₁_ = combined MIC _A₁_/alone MIC _A₁_ and FICI _B =_ combined MIC _B₂_/alone MIC _B₁_. The results were interpreted as synergism (FICI ≤ 0.5), addition (0.5 ≤ FICI ≤ 1), indifference (FICI ≤ 4), or antagonism (FICI ≥ 4).

### Flow cytometry

Suspensions containing approximately 5 × 10⁴ CFU/mL of *Alicyclobacillus* strains, treated or not with 1× MIC of D-limonene, nisin, or their combination, were incubated at 45 °C for 24 h in BAT broth. After incubation, the cultures were centrifuged (5000 × g, 10 min), washed twice with 0.85% saline solution, and resuspended in the same solution. Cell viability was assessed using the BacLight™ LIVE/DEAD^®^ Bacterial Viability and Counting Kit (Invitrogen, Thermo Fisher Scientific, Eugene, OR, USA). For staining, 1 mL of each cell suspension was mixed with SYTO 9 (final concentration 3.34 µM) and propidium iodide (final concentration 20 µM) and incubated in the dark for 15 min at room temperature.

Acquisition was performed on a BD FACSCanto™ II flow cytometer using the FITC (SYTO 9) and PE (PI) channels. A minimum of 10,000 events per sample was collected. Forward and side scatter parameters were used to gate bacterial populations and exclude debris. Viability was calculated based on the proportion of SYTO 9–positive/PI–negative cells relative to untreated controls. Heat-killed cells (70 °C, 30 min) were used as dead controls to define gating boundaries [23].

### Test in orange juice

For the food matrix assay, concentrated orange juice, donated by an industrial juice producer located in the Paranavaí/Porto Rico region (Brazil), was reconstituted to 11 °Brix. Before inoculation, the juice was verified to be free of *Alicyclobacillus* spp. and other contaminants by plating 1 mL aliquots on BAT agar, followed by incubation at 45 °C for 72 h to ensure that even slow-growing strains or damaged spores were detected. Subsequently, 100 µL aliquots of the reconstituted juice were dispensed into 96-well microplates (TPP^®^, Switzerland). The assays were performed separately for vegetative cells and spores of *A. acidoterrestris*. The inoculum was prepared to achieve a final concentration of 5 × 10⁴ CFU/mL (for vegetative cells) or spores/mL (for the spore suspension), and treated with D-limonene, nisin, and their combination at concentrations ranging from 1000 to 0.0001 µg/mL. After incubation at 45 °C for 24 h, the survival of cells and spores was determined by plating on BAT agar. To allow for the recovery of sub-lethally injured cells and germination of treated spores in the food matrix, the plates were incubated for up to 72 h before final colony counting. All tests were conducted in triplicate [23].

### Volatile profile analysis (Electronic Nose)

Orange juice samples (Control, DL, nisin, and the DL+nisin combination), treated with concentrations equivalent to the MEC, were analyzed in duplicate. The 10 mL aliquots were transferred to 40 mL screw-capped bottles with silicone septa and analyzed in duplicate. The bottles were kept in a desiccator (Nalgon, model 0880) at 35 °C for 30 min to form the static headspace (SH). The analysis of volatile organic compounds (VOCs) was performed according to a method adapted from Vieira et al. [24] using a homemade electronic nose Makimori e Bona et al. [25] equipped with metal oxide semiconductor (MOS) sensors, models MQ2, MQ3, MQ8, MQ9, MQ137, and MQ138 (Hanwei Electronics). The sampling parameters were: capsule at 25 °C; initial purge of 60 s; sampling time of 30 s (with baseline); final purge of 60 s; and pump flow rate of 4 L/min. Data acquisition was performed using Enose CAD 2.3 software [26]. The sensor signals were combined into a single matrix and subjected to principal component analysis (PCA) and hierarchical cluster analysis (HCA). The area under the curve of each sensor was calculated, and the samples were statistically compared using Tukey ‘s test, with analysis conducted in MATLAB R2023a software [27].

### Antioxidant activity of D-limonene

For the evaluation of antioxidant activity (AA), the ABTS (2,2’-azino-bis-3-ethylbenzothiazoline-6-sulfonic acid) radical scavenging method and the ferric reducing antioxidant power (FRAP) method were used. In the ABTS (AA-ABTS) method, a 7 mM ABTS (Sigma Aldrich) solution was prepared and subsequently diluted to an absorbance of 0.70 ± 0.05 at 734 nm. After the addition of the extracts, the mixture was left to stand for 6 min, and the absorbance was measured again at 734 nm, as described by [28].

In the FRAP assay (AA-FRAP), 90 µL of the extract, diluted in 0.27 mL of distilled water, was added to 2.7 mL of FRAP reagent (Sigma Aldrich) (0.3 mM), composed of 25 mL of 0.3 mM acetate buffer solution (Sigma Aldrich) (pH 3.6), 2.5 mL of 10 mM TPTZ (2,4,6-tri(2-pyridyl)−1,3,5-triazine, Sigma Aldrich) solution in 40 mM HCl, and 2.5 mL of 20 mM ferric chloride (Sigma Aldrich). The mixture was incubated at 37 °C for 30 min, and the absorbance was read at 595 nm, according to [29].

Quantification of antioxidant activity was performed based on analytical curves obtained from standard solutions of Trolox (6-hydroxy-2,5,7,8-tetramethylchromano-2-carboxylic acid, Sigma Aldrich), in concentration ranges of 0.10 to 0.80 mM for AA-FRAP and 0.20 to 2.0 mM for AA-ABTS. The results were expressed in µmol of Trolox equivalent (TE) per 100 g.

### Scanning electron microscopy (SEM)

Samples containing *Alicyclobacillus* spp. treated with D‑limonene, nisin, and the DL + Nis combination were initially washed with saline solution and fixed in 2.5% glutaraldehyde (Sigma‑Aldrich) in 0.1 M sodium cacodylate buffer (Sigma‑Aldrich). After fixation, a second wash was performed with the same buffer, followed by coating with poly-L-lysine. The dehydration process was conducted with increasing series concentrations of ethanol from 50% to 100%, with each step lasting 10 min, and finalized by critical point drying with carbon dioxide (CO₂). Subsequently, the samples were metallized with gold and examined by scanning electron microscopy (SEM, Quanta 250; FEI Company) [30].

### Statistical analysis

All assays were performed in at least three independent experiments, each carried out in triplicate. The results are expressed as the mean of these independent replicates ± standard deviation. The data were subjected to analysis of variance (ANOVA), followed by Tukey ‘s test (p < 0.05) for comparison of means. Statistical analyses were performed using Statistica^®^ software version 10.0 (StatSoft Inc).

## Results and discussion

### Antibacterial activity by disk diffusion

The results for antimicrobial activity (Table 1) show that all treatments had inhibitory activity against the five strains of *Alicyclobacillus* tested.

**Table 1:** Antibacterial activity of D-limonene (DL), Nisin (Nis), and combination (DL + Nis) in disk diffusion assays

Treatment	Concentration ul/disc	A. acidoterrestris 0244^T^	A. herbarius 0246^T^	A. acidiphilus 0247^T^	A. hesperidum 0298^T^	A. sendaiensis KCTC 3843^T^
DL	20	11 ^Ca^ ± 0.50	10 ^Ca^ ± 0.50	10 ^Ca^ ± 0.50	10 ^Ca^ ± 0.50	10 ^Ca^ ± 0.50
Nis	20	21 ^Bb^ ± 0.50	22 ^Bb^ ± 0.50	22 ^Bb^ ± 0.50	24 ^Ba^ ± 0.50	20 ^Bb^ ± 0.50
DL + Nis	20	24 ^Aa^ ± 0.50	25 ^Aa^ ± 0.50	26 ^Aa^ ± 0.50	26 ^Aa^ ± 0.50	26 ^Aa^ ± 0.50
Control	0	0	0	0	0	0

The diameter (mm) of the inhibition zone at a concentration of 20 µL/disc is the mean of three independent experiments, including the paper disc diameter (6 mm). Mean values followed by the same uppercase letters in the column and lowercase letters in the row do not differ statistically according to Tukey’s test (*p* > 0.05)

When tested in isolation, DL exhibited inhibitory activity, with inhibition halos ranging from 10 mm to 11 mm. Li et al. [31] also observed similar results when evaluating isolated D-limonene, with average inhibition halos of approximately 11 mm against *Escherichia coli*, *Salmonella*, and *Lactobacillus acidophilus*, confirming its antibacterial activity.

Nis presented significantly larger inhibition halos (*p* < 0.05) than those observed for DL, with diameters ranging from 20 mm to 24 mm. The most sensitive strain to this treatment was *A. hesperidum* (24 ± 0.5 mm), and the least sensitive was *A. sendaiensis* (20 ± 0.5 mm), evidencing a variation in susceptibility among the species.

When combined, DL and Nis resulted in a significant increase (*p* < 0.05) in the inhibition halo for all strains compared to the isolated treatments, which ranged from 24 mm for *A. acidoterrestris* to 26 mm for the strains *A. acidiphilus*,* A. hesperidum*, and *A. sendaiensis*. Compared to DL alone, the combination promoted a 160% increase in the diameter of the inhibition halo for *A. acidiphilus*,* A. hesperidum.* and *A. sendaiensis*; 150% for *A. herbarius* and 118% for *A. acidoterrestris*. Regarding Nis, the combination showed a percentage increase in the halo, being 30.0% for *A. sendaiensis*, 18.2% for *A. acidiphilus*, 14.3% for *A. acidoterrestris*, 13.6% for *A. herbarius.* and 8.3% for *A. hesperidum*. These results indicate a synergistic interaction between the two compounds against the genus. *Alicyclobacillus* spp.

Species of the genus *Alicyclobacillus*, such as *A. acidoterrestris*,* A. herbarius*,* A. acidiphilus*,* A. hesperidum A. sendaiensis* and other spore-forming microorganisms are among the main spore-forming microorganisms associated with juice spoilage. Among them, *A. acidoterrestris* is considered the most relevant due to its high incidence of contamination in industrial orange juice processes [5].

### Minimum inhibitory concentration (MIC) and minimum bactericidal concentration (MBC)

The MIC and MBC values, presented in Table 2, allowed us to evaluate the effectiveness of each treatment against the species of *Alicyclobacillus*. The DL demonstrated bactericidal activity against all strains, with MBC values being identical to the MIC. However, the lower MIC values (62.5 µg/mL) observed for *A. acidiphilus* and *A. hesperidum* showed greater sensitivity compared to the others, which required concentrations of 125 µg/mL for inhibition. Nis, in turn, demonstrated greater antibacterial efficacy than DL, with distinct sensitivity among the species, being *A. hesperidum* the most susceptible (MIC 7.81 µg/mL), whereas *A. herbarius* and *A. sendaiensis* were the least susceptible (MIC 31.25 µg/mL). The effect of Nis was consistently bactericidal for all five species tested, with MBC values being equal to or double the MIC values.

**Table 2:** Minimum inhibitory concentration (MIC) and minimum bactericidal concentration (MBC) of D-limonene (DL), nisin (Nis), and combination (DL + Nis)

Parameters	Species	DL (µg/mL)	Nis (µg/mL)	DL + Nis (µg/mL)
MIC	*A. acidoterrestris* 0244^T^	125 ^Bc^ ± 0.00	15.62 ^Bb^± 0.00	1.95 ^Ba^ ± 0.00
	*A. herbarius* 0246^T^	125 ^Bc^ ± 0.00	31.25 ^Cb^ ± 0.00	3.90 ^Ca^ ± 0.00
	*A. acidiphilus* 0247^T^	62.5 ^Ac^ ± 0.00	15.62 ^Bb^ ± 0.00	0.12 ^Aa^ ± 0.00
	*A. hesperidum* 0298^T^	62.5 ^Ac^ ± 0.00	7.81 ^Ab^ ± 0.00	0.12 ^Aa^ ± 0.00
	*A. sendaiensis* KCTC 3843^T^	125 ^Bc^ ± 0.00	31.25 ^Cb^ ± 0.00	1.95 ^Ba^ ± 0.00
MBC	*A. acidoterrestris* 0244^T^	125 ^Bc^ ± 0.00	15.62 ^Ab^ ± 0.00	1.95 ^Ba^ ± 0.00
	*A. herbarius* 0246^T^	125 ^Bc^ ± 0.00	31.25 ^Bb^ ± 0.00	3.90 ^Ca^ ± 0.00
	*A. acidiphilus* 0247^T^	62.5 ^Ac^ ± 0.00	15.62 ^Ab^ ± 0.00	0.12 ^Aa^ ± 0.00
	*A. hesperidum* 0298^T^	62.5 ^Ac^ ± 0.00	15.62 ^Ab^ ± 0.00	0.12 ^Aa^ ± 0.00
	*A. sendaiensis* KCTC 3843^T^	125 ^Bc^ ± 0.00	31.25 ^Bb^ ± 0.00	1.95 ^Ba^ ± 0.00

Mean values followed by the same uppercase letters in the column and lowercase letters in the row do not differ statistically according to Tukey’s test (*p* > 0,05)

The combination of DL + Nis showed the most effective antibacterial effect among the treatments. The MIC values were lower for all strains, ranging from 0.12 µg/mL to 3.90 µg/mL, which demonstrates a synergistic interaction. Similar to the DL or Nis treatments, the combination also revealed a consistently bactericidal effect, with MBC values identical to the MIC. This result indicates that the synergy not only intensifies the inhibitory action but also overcomes the individual limitations of the compounds, such as the lower activity of D-limonene and the variability in response to nisin among different strains of *Alicyclobacillus*.

This synergism becomes evident when analyzing the reduction in MIC. Compared to DL alone, the combination was 521 times more potent against *A. acidiphilus* and *A. hesperidum*; 64 times more potent against *A. acidoterrestris* and *A. sendaiensis;* and 32 times more potent against *A. herbarius*. Even in relation to Nis, the combination was notably superior, being 130 times more effective against *A. acidiphilus*; 65 times more effective against *A. hesperidum;* 16 times more effective against *A. sendaiensis;* and 8 times more effective against *A. acidoterrestris* and *A. herbarius*.

The MIC and MBC values obtained in this study for nisin are in agreement with the results described by Ruiz et al. [32]. Furthermore, Oliveira et al. [33] observed that the combination of nisin with *Matricaria chamomilla* L. showed a synergistic effect, potentiating the antimicrobial action of both compounds. The nisin mechanism of action, already well established in the literature, occurs through the formation of pores in the cell membrane, causing loss of intracellular content and cell death [21]. D-limonene, due to its lipophilic nature, acts as a destabilizing agent of the bacterial cell membrane, increasing its permeability. This effect facilitates the penetration of nisin into the cell, potentiating its action on lipid targets and resulting in more effective microbial inhibition [17, 34].

### Synergism analysis using checkerboard

**Fig. 1:**
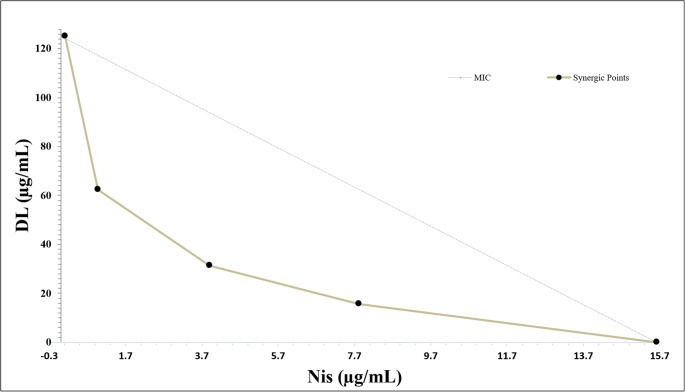
Isobologram in*A. acidoterrestris* between D-limonene (DL) 125 µg/mL and nisin (Nis) 15.7 µg/mL (FICI= 0.07)

The interaction between DL and Nis against *A. acidoterrestris* showed an FIC of 0.07. According to the established criteria, FIC values ≤ 0.5 indicate a synergistic interaction between the two compounds. This synergy is visually represented in the isobologram in Fig. 1. The graph was constructed by plotting the individual MICs of Nis and DL on the x and y axes, respectively, with a diagonal line representing theoretical additivity (FICI = 1.0).The iso-effectiveness curve for the combination is positioned significantly below this additivity line, confirming that the combined effect is greater than the sum of the individual effects.

The synergism observed in the present study is aligned with previous works that investigated the combination of nisin with compounds of natural origin [35, 36]. For example, Oliveira et al. [33] evaluated the combined action of nisin with chamomile extract against bacteria of the genus *Alicyclobacillus*, and observed a synergistic effect with FIC index of 0.068.

Zhang et al. [17] also observed synergistic and additive effects of the association between nisin and D-limonene against foodborne microorganisms, with FIC values of 0.37 for *Staphylococcus aureus* and *Bacillus subtilis* (synergism), 0.50 for *Saccharomyces cerevisiae*, and 1.00 for *Escherichia coli* (useful additive effect), with no record of antagonistic or indifferent effects. The findings of the present study reinforce the potential of the DL + Nis combination as an effective antibacterial strategy against *A. acidoterrestris*.

### Minimum sporicidal concentration (MSC)

The effectiveness of the treatments was evaluated in reconstituted orange juice against vegetative cells and spores of *A. acidoterrestris*. The corresponding results are presented in Table 3. Spores proved to be more resistant than vegetative cells in all treatments. However, the synergistic combination of DL and Nis was effective against both treated forms. Against vegetative cells, all treatments differed from each other (*p* < 0.05), with the DL + Nis combination being the most effective (lowest MSC), 0.0009 µg/mL (0.9 ng/mL), followed by Nis (0.01 µg/mL) and DL (3.9 µg/mL). This order of effectiveness was maintained against spores, but at considerably higher concentrations.

**Table 3:** Minimum inhibitory concentration (MIC) and Minimum sporicidal concentration (MSC) in orange juice for *A. acidoterrestris*

Parameters	Vegetative cells A. acidoterrestris 0244^T^	Spores A. acidoterrestris 0244^T^
DL (µg/mL)	3.9 ^Ca^ ± 0.00	62.5 ^Cb^ ± 0.00
Nis (µg/mL)	0.01 ^Ba^ ± 0.00	7.81 ^Ba^ ± 0.00
DL + Nis (µg/mL)	0.0009 ^Aa^ ± 0.00	0.48 ^Aa^ ± 0.00

Mean values followed by the same uppercase letters in the row and lowercase letters in the columns do not differ statistically according to Tukey’s test (*p* > 0.05). *D-limonene (DL), nisin (Nis), and combination (DL + Nis)

DL + Nis combination inactivated spores at 0.48 µg/mL, a lower value (*p* < 0.05) than that of nisin (7.81 µg/mL) and D-limonene (62.5 µg/mL). This result proves to be efficient when compared to other synergistic studies, such as that of Oliveira et al. [33] which, using a combination of *Matricaria chamomilla* L. extract with nisin obtained a MSC of 7.81 µg/mL against *A. acidoterrestris* spores. The spore’s resistance is evident when comparing the concentrations necessary for their inactivation with those of vegetative cells, which demand approximately 16 times more DL, 780 times more Nis, and 530 times more of the combination to inactivate the spores. Despite the high intrinsic resistance of the spores, the result demonstrates that the synergistic interaction remains effective, being able to inactivate them at a very low concentration (0.48 µg/mL) in orange juice. These results are relevant because the survival and germination of spores after thermal processing is the main challenge in the control of *Alicyclobacillus* in the juice industry [4].

Lin et al. [34] state that the effect of D-limonene is due to the rupture of cell membranes and walls, caused by its lipophilic properties. According to Bertuso et al. [37] the effectiveness in inactivating spores may be associated with the lipophilic and amphiphilic properties of certain compounds, such as biosurfactants and essential oils. These characteristics favor interaction with the structures of the spore coating, promoting its rupture and hindering germination. Furthermore, the hydrophilic-lipophilic balance (HLB) has been related to the ability to penetrate and destabilize the inner membrane, especially in more hydrophobic compounds [38].

In this context, the synergistic effect observed for the DL + Nis combination may be explained by a complementary mechanism of action. DL, due to its strong lipophilicity, likely disrupts the outer layers of the spore and increases the permeability of the inner membrane, facilitating the access of nisin to lipid II during early germination events. Nisin, in turn, accelerates membrane permeabilization through pore formation, leading to leakage of intracellular content and loss of viability. This hypothesis is consistent with previous synergistic studies involving nisin and natural compounds [34, 37] and provides a plausible explanation for the markedly lower MSC observed for the combination.

### Cellular morphology by sem and flow cytometry

**Fig. 2:**
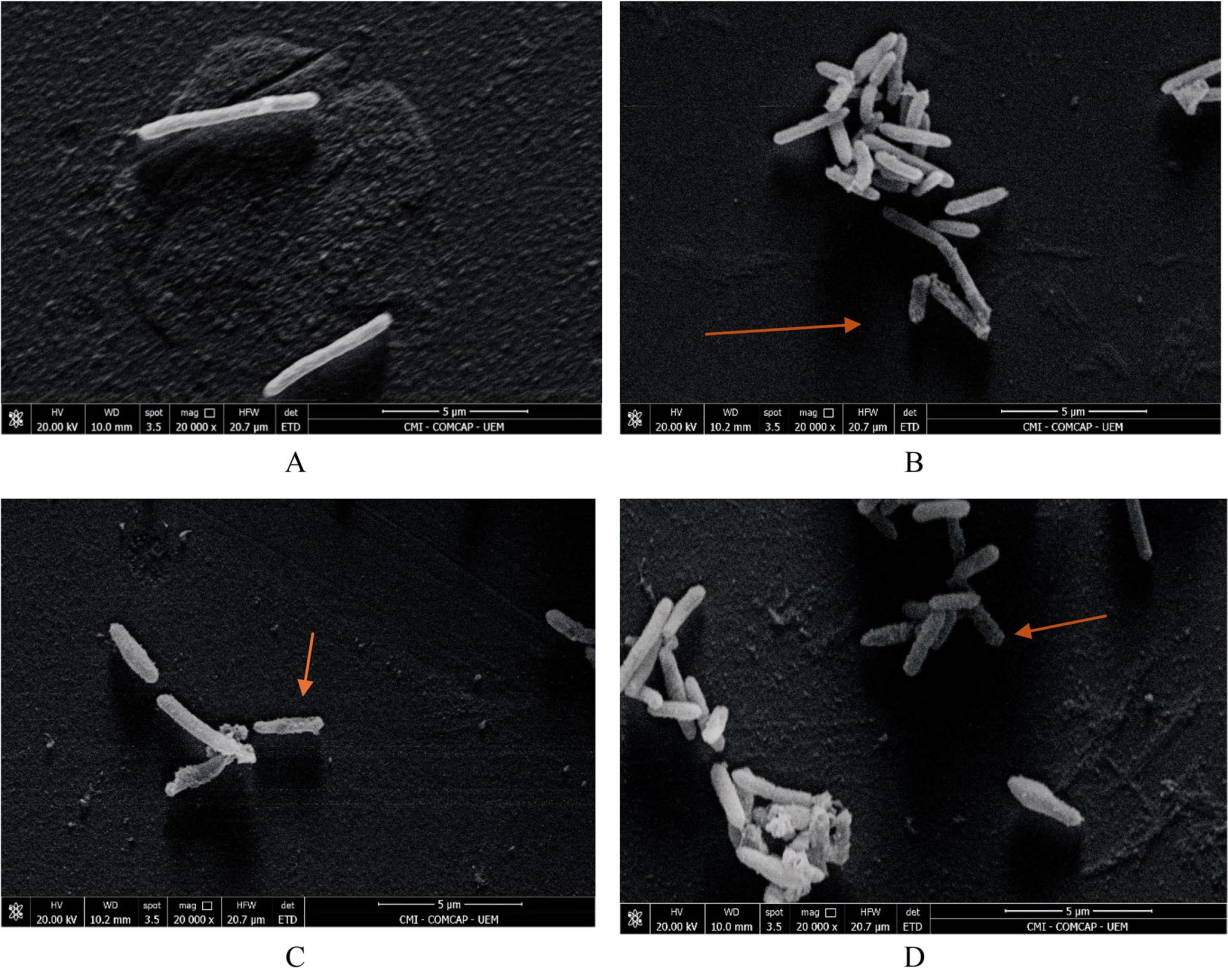
Scanning electron microscopy analysis of *A. acidoterrestris* vegetative cells. Cells were exposed for 24 h to subinhibitory concentrations of treatments. (**A**) Control cells, with intact morphology; (**B**) Cells treated with D-limonene (DL), showing surface wrinkling; (**C**) Cells treated with nisin (Nis), indicating significant surface depression and potential loss of membrane integrity; (**D**) Cells treated with the combination (DL+Nis), severe structural collapse and cellular debris, consistent with cell lysis. Arrows indicate main morphological damages. Scale bar = 5 µm. Magnification = 20,000x

The micrographs obtained (Fig. 2) illustrate the morphological alterations in vegetative cells of *A. acidoterrestris* following exposure to subinhibitory concentrations of D-limonene (DL), nisin (Nis), and their combination (DL + Nis). Control cells (Fig. 2a) displayed a typical bacillary morphology with smooth surfaces and regular contours, indicating intact structural integrity. Cells treated with DL (Fig. 2b) exhibited distorted and irregular surfaces. This pattern is consistent with the lipophilic nature of D-limonene, which accumulates in the lipid bilayer, increasing membrane fluidity and promoting structural destabilization [17].

Treatment with nisin (Fig. 2c) revealed notable surface depressions and localized deformations. While nisin is known for its ability to bind to Lipid II and form transmembrane pores [5, 39], at the resolution provided by SEM, these alterations appear as significant morphological collapses. The DL + Nis combination (Fig. 2d) resulted in severe structural damage, characterized by profound cellular shrinkage and the presence of extracellular debris. Although SEM does not allow confirmation of functional alterations, the visual findings corroborate the quantitative data from the checkerboard assay (FICI = 0.07), suggesting synergism between the compounds. The hypothesis is that DL compromises membrane integrity, facilitating the action of nisin, which may explain the high efficacy of the combination even at low concentrations.

**Fig. 3:**
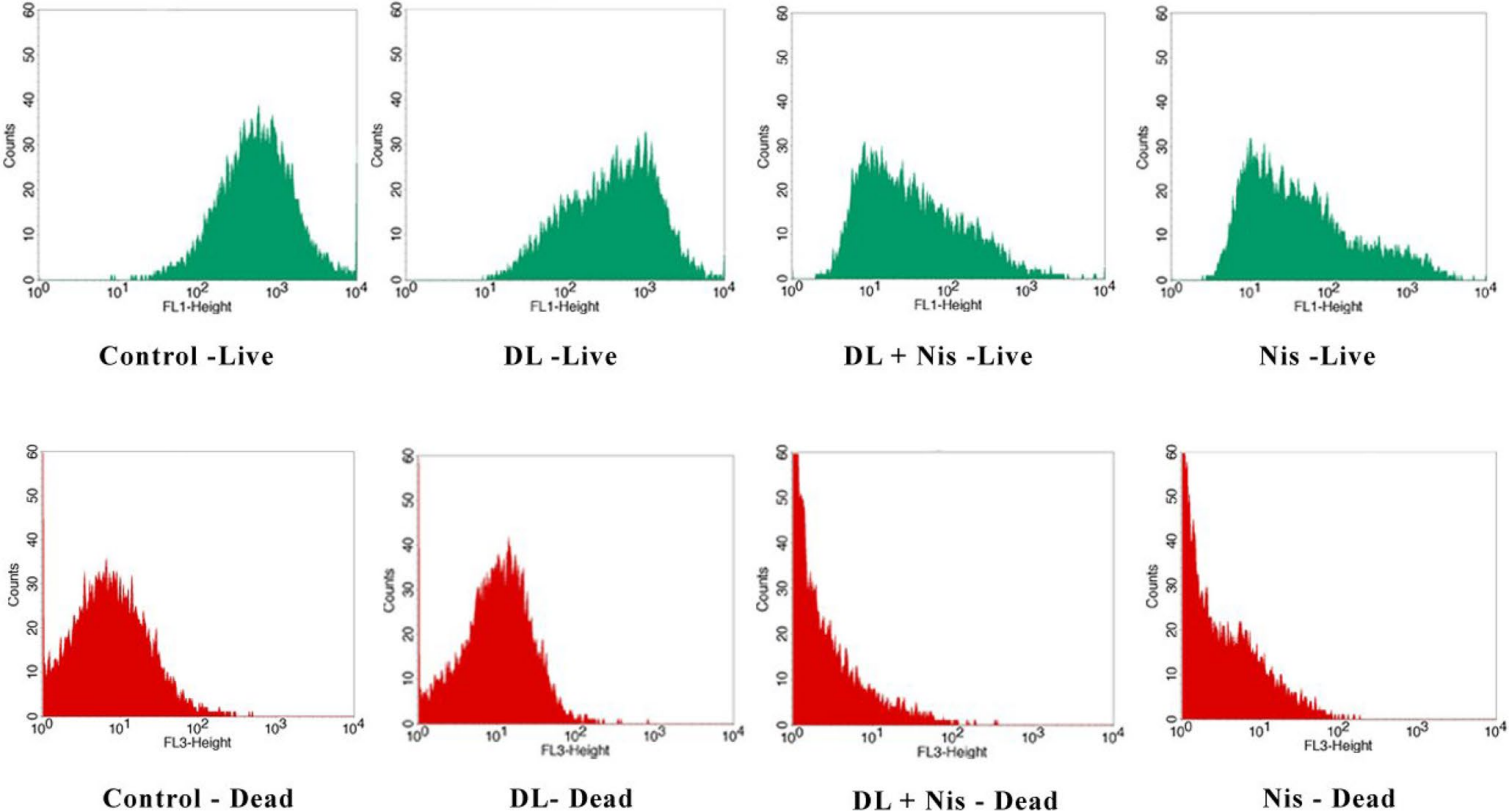
Effects of D-limonene (DL) and Nisin (Nis), isolated and in combination (DL+Nis), on the viability of *A. acidoterrestris* assessed by flow cytometry. Top row: fluorescence channel 1 (FL1) histograms, detecting SYTO 9 dye (Live). Bottom row: fluorescence channel 3 (FL3) histograms, detecting Propidium Iodide dye (Dead)

In parallel with these morphological observations, the functional integrity of the membrane was evaluated by flow cytometry (Fig. 3). Control cells presented a high viability profile, characterized by high fluorescence intensity in the FL1 channel (SYTO 9-positive) and low intensity in the FL3 channel (PI-negative).

Conversely, DL treatment confirmed membrane damage, evidenced by a significant increase (*p* < 0.01) in PI uptake, visualized by the shift of the population toward the FL3-high quadrant. This confirms that the morphological distortions seen in SEM correspond to a physical loss of the membrane barrier function.

Notably, the treatments with Nis and the DL + Nis combination caused a drastic reduction in fluorescence intensity in both FL1 and FL3 channels, shifting the population toward the lower-left quadrant, typical of cellular autofluorescence. This phenomenon is a strong indicator of extensive cell lysis; when cells undergo severe fragmentation, the leakage of intracellular contents and the lack of intact nucleic acids prevent effective staining by the fluorophores. These functional data reinforce the hypothesis of a lytic synergism: DL likely destabilizes the protective outer layers, facilitating the access and pore-forming activity of nisin, leading to total cellular collapse even at low concentrations [33].

### Sensory profile analysis (E-Nose) and antioxidant activity (AA)

**Fig. 4:**
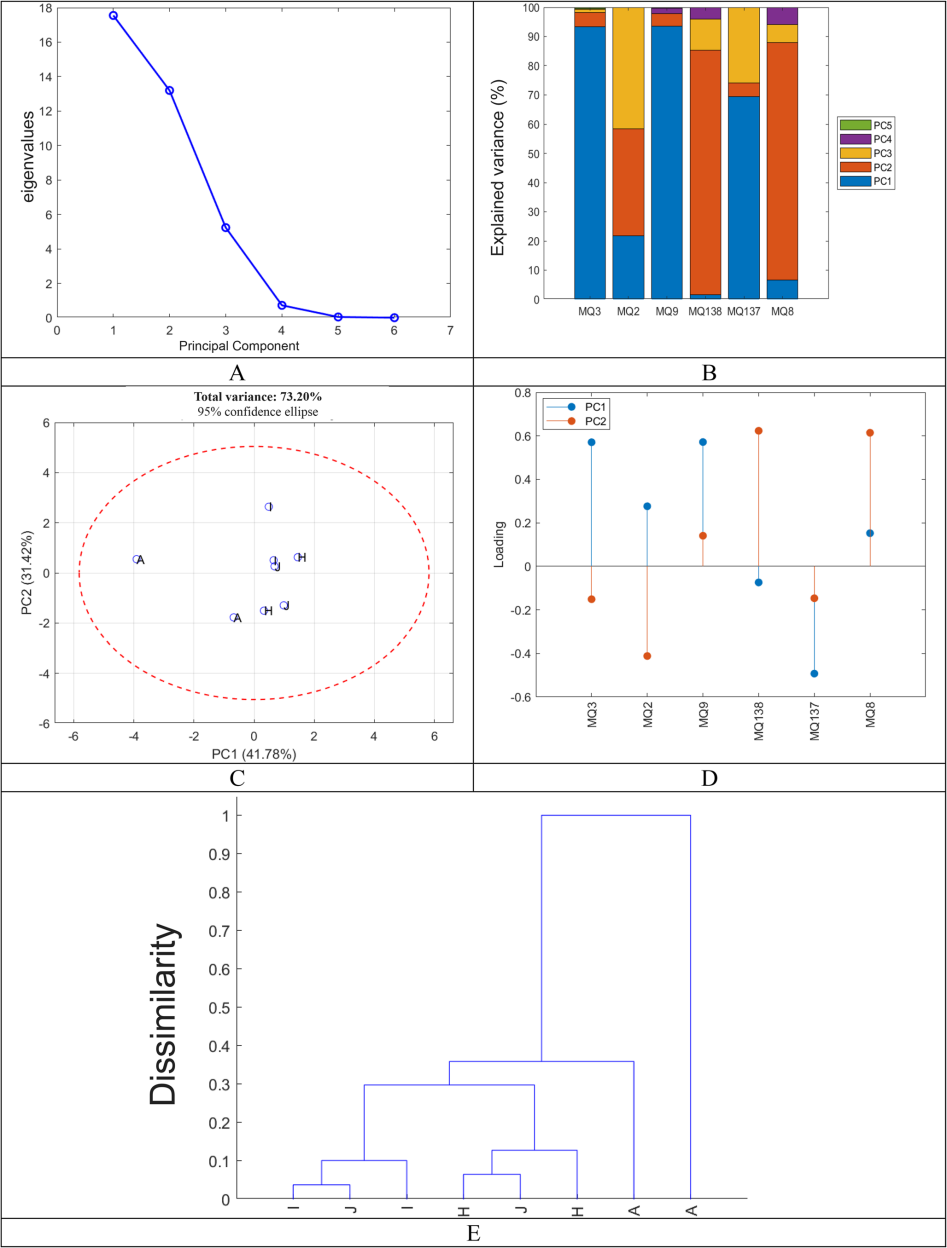
Multivariate and univariate analysis of the volatile profile of orange juice samples. **a** Scree plot showing the eigenvalues of the principal components; **b** Extracted variance by each component; **c** Principal Component Analysis (PCA) score plot of PC1 versus PC2; **d** Loading plot of the variables; **e** Hierarchical Cluster Analysis (HCA) dendrogram showing the dissimilarity among treatments. *Control (A), D-limonene (H), nisin (I), and the combination (J)

The volatile profile analysis aimed to evaluate the sensory impact of adding the antibacterial compounds to orange juice. The test sought to determine if DL, Nis, and their combination (DL + Nis), at previously established minimum sporicidal concentrations (MSCs), would alter the characteristic aroma of the product. For this, analyses were performed on pure orange juice (Control-A), without microbial contamination, comparing it with juice to which the treatments were added (DL-H; nisin-I; DL+nisin-J). Principal Component Analysis (PCA) was applied to the maximum intensity data from the sensors to explore the variance between samples in Fig. 4.

The PCA model was evaluated using a scree plot (Fig. 4a), indicating that the first three principal components (PCs) were the most relevant, with PC1 explaining 41.78%, PC2 31.42%, and PC3 12.46% of the data variance. The variance analysis (Fig. 4b) showed that PC1 was primarily associated with sensors MQ3, MQ9, and MQ137 (> 70% variance), while PC2 was linked to sensors MQ2, MQ138, and MQ8 (36.5%, 83.7%, and 81.3% variance, respectively). PC3 was excluded from graphical interpretation due to its low contribution to total variance.

The score plot (Fig. 4c) showed an absence of defined clusters, with samples concentrated near the origin, indicating high similarity between the groups. The minor separations observed are attributed to inherent random variations rather than systematic differences, making an in-depth loadings discussion (Fig. 4d) unnecessary. Consequently, no relevant differences were observed in the volatile profile of samples H, I, and J compared to the control. These findings were confirmed by univariate statistical analysis (Table S1, Supplementary Material), showing no significant difference (*p* > 0.05) among treatments for five of the six sensors, with only MQ137 showing a slight variation in the control.

Furthermore, Hierarchical Cluster Analysis (Fig. 4e), using Euclidean metric and Ward’s method, confirmed this high similarity. The dendrogram showed treated samples forming an initial group with 70.2% similarity, which then merged with the control duplicates, indicating the absence of a robust distinction between groups.

Taken together, the results of the multivariate and univariate analyses demonstrate that the addition of D-limonene, nisin, and their combination did not cause a significant alteration in the volatile profile of orange juice. This finding is of great practical relevance because, according to Ferreira et al. [21], the industrial viability of a new preservative depends both on its microbiological efficacy and its ability to avoid introducing “off-flavors” to the product. The E-nose approach to evaluate natural antimicrobials is corroborated by other studies. Oliveira [33], for example, used E-nose to assess the sensory impact of combining nisin and *Matricaria chamomilla* L. extract and detected changes in the volatile profile, suggesting that D-limonene may represent a more sensorially neutral alternative. On the other hand, Gobbi et al. [40] employed the E-nose as an early diagnostic tool for the detection of *Alicyclobacillus* spp. in juices, demonstrating its effectiveness in identifying volatile changes associated with microbial contamination. This application highlights the potential of sensory monitoring, whether instrumental or perceptual, as a complementary strategy in the quality control of acidic beverages.

In addition to the exemplified sensory neutrality, the antioxidant capacity of D-limonene evaluated by the ABTS (0.23 µmol Trolox/100 g) and FRAP (91.67 µmol Trolox/100 g) methods suggests a secondary benefit. Sensory deterioration in juices is not only caused by microorganisms, but also by the oxidation of volatile compounds. The antioxidant activity of D-limonene [41, 42] may protect oxidation-sensitive components, contributing to the preservation of the original sensory attributes of the juice [17].

Thus, the data indicate that the DL + Nis combination not only controls *Alicyclobacillus*, preventing microbial “off-flavors” such as guaiacol, but also provides antioxidant protection to the juice, reducing oxidative degradation and the resulting chemical off-flavors. Additionally, the antioxidant activity of D-limonene is recognized for its benefits to consumer health, such as the neutralization of reactive oxygen species [34].

## Conclusion

In conclusion, the DL + Nis combination represents a promising natural alternative for controlling *Alicyclobacillus* spp. in orange juice. While the results demonstrate high efficacy and sensory neutrality in a laboratory-scale food matrix, further studies on long-term stability and large-scale industrial feasibility are necessary to fully establish its commercial application. Furthermore, the application of these natural compounds aligns with the ‘clean label’ trend, meeting consumer demand for beverages free from synthetic additives while maintaining sensory quality and microbial safety [43].

## Supplementary Information

Below is the link to the electronic supplementary material.

Supplementary Material 1 (DOCX 16.2 KB)

## Data Availability

The data will be available upon request.

## References

[1] Wang R, Thammasuwan R, Roth K, Tongchitpakdee S, Worobo R (2025) Control of *Alicyclobacillus acidoterrestris* in apple juice with natural antimicrobial glycolipid. J Food Prot 88(3):100460. 10.1016/j.jfp.2025.100460 39900181 10.1016/j.jfp.2025.100460

[2] Snyder AB, Martin N, Wiedmann M (2024) Microbial food spoilage: impact, causative agents and control strategies. Nat Rev Microbiol 22(9):528–542. 10.1038/s41579-024-01037-x 38570695 10.1038/s41579-024-01037-x

[3] CitrusBR (2024) Produção de laranjas e suco de laranja na safra 2024/25. São Paulo: Associação Nacional dos Exportadores de Sucos Cítricos. https://citrusbr.com/imprensa/releases/2024 *(accessed 15 October 2024).*

[4] Dutra TV, de Menezes JL, Mizuta AG, de Oliveira A, Moreira TFM, Barros L, Mandim F, Pereira C, Gonçalves HO, Leimann FV, Mikcha JMG, Junior MM, Abreu Filho BA (2021) Use of nanoencapsulated curcumin against vegetative cells and spores of *Alicyclobacillus* spp. in industrialized orange juice. Int J Food Microbiol 360:109442. 10.1016/j.ijfoodmicro.2021.109442 34688124 10.1016/j.ijfoodmicro.2021.109442

[5] Pornpukdeewattana S, Jindaprasert A, Massa S (2020) *Alicyclobacillus* spoilage and control—a review. Crit Rev Food Sci Nutr 60(1):108–122. 10.1080/10408398.2018.1516190 30729793 10.1080/10408398.2018.1516190

[6] Han Y, Sun Z, Chen W (2019) Antimicrobial susceptibility and antibacterial mechanism of limonene against *Listeria monocytogenes*. Molecules 25(1):33. 10.3390/molecules25010033 31861877 10.3390/molecules25010033 PMC6982812

[7] Pascoli IC, dos Anjos MM, da Silva AA, Lorenzetti FB, Cortez DAG, Mikcha JMG, Abreu Filho BA (2018) Piperaceae extracts for controlling *Alicyclobacillus acidoterrestris* growth in commercial orange juice. Ind Crops Prod 116:224–230. 10.1016/j.indcrop.2018.02.073

[8] Menezes JL, Mizuta AG, Dutra TV, Ferreira TV, Bonin E, Castro JC, Schipfer CWT, Szczerepa MMA, Lancheros CAC, Pilau EJ, Machinski Junior M, Mikcha JMG, Abreu Filho BA (2022) Kefir fermented fruit by-products: anti-*Alicyclobacillus* spp. activity and antioxidant activity. Food Sci Technol 42:e117621. 10.1590/fst.117621

[9] Mizuta AG, de Menezes JL, Dutra TV, Ferreira TV, Castro JC, da Silva CAJ, Pilau EJ, Junior MM, Abreu Filho BA (2020) Evaluation of antimicrobial activity of green tea kombucha at two fermentation time points against Alicyclobacillus spp. LWT 130:109641. 10.1016/j.lwt.2020.109641

[10] Anjos MM, Endo EH, Leimann FV, Gonçalves OH, Dias-Filho BP, Abreu Filho B (2018) Preservation of the antibacterial activity of enzymes against *Alicyclobacillus* spp. through microencapsulation. LWT 88:18–25. 10.1016/j.lwt.2017.09.039

[11] Food US, Administration D (2025) Substances Added to Food (formerly EAFUS): D-Limonene. Retrieved from FDA database *(accessed 14 October 2025).* https://www.hfpappexternal.fda.gov/scripts/fdcc/index.cfm?set=FoodSubstances *&id=LIMONENED*

[12] U.S. Food and Drug Administration. 21 CFR § 184.1538 — Nisin preparation. Accessed 14 October (2025) https://www.ecfr.gov/current/title-21/chapter-I/subchapter-B/part-184/subpart-B/section-184.1538

[13] Field D, Fernandez de Ullivarri M, Ross RP, Hill C (2023) After a century of nisin research – where are we now? FEMS Microbiol Rev 47:fuad023. 10.1093/femsre/fuad023 37300874 10.1093/femsre/fuad023 PMC10257480

[14] Sourri P, Tassou CC, Nychas GJE, Panagou EZ (2022) Fruit juice spoilage by *Alicyclobacillus*: detection and control methods—a comprehensive review. Foods 11(5):747. 10.3390/foods11050747 35267380 10.3390/foods11050747 PMC8909780

[15] Campion A, Morrissey R, Field D, Cotter PD, Hill C, Ross RP (2017) Use of enhanced nisin derivatives in combination with food-grade oils or citric acid to control *Cronobacter sakazakii* and *Escherichia coli* O157:H7. Food Microbiol 65:254–263. 10.1016/j.fm.2017.01.020 28400011 10.1016/j.fm.2017.01.020

[16] Zhao X, Shi C, Meng R, Liu Z, Huang Y, Zhao Z, Guo N (2016) Effect of nisin and perilla oil combination against Listeria monocytogenes and Staphylococcus aureus in milk. J Food Sci Technol 53:2644–2653. 10.1007/s13197-016-2236-6 27478220 10.1007/s13197-016-2236-6 PMC4951417

[17] Zhang Z, Vriesekoop F, Yuan Q, Liang H (2014) Effects of nisin on the antimicrobial activity of D-limonene and its nanoemulsion. Food Chem 150:307–312. 10.1016/j.foodchem.2013.10.160 24360455 10.1016/j.foodchem.2013.10.160

[18] Deinhard G, Blanz P, Poralla K, Altan E (1987) *Bacillus acidoterrestris* sp. nov., a new thermotolerant acidophile isolated from different soils. Syst Appl Microbiol 10(1):47–53. 10.1016/S0723-2020(87)80009-7

[19] Prado DB, Szczerepa MMA, Capeloto OA, Astrath NGC, Santos NCA, Previdelli ITS, Nakamura CV, Mikcha JMG, Abreu Filho BA (2019) Effect of ultraviolet (UV-C) radiation on spores and biofilms of *Alicyclobacillus* spp. in industrialized orange juice. Int J Food Microbiol 305:108238. 10.1016/j.ijfoodmicro.2019.108238 31174101 10.1016/j.ijfoodmicro.2019.108238

[20] CLSI (2018) Methods for Dilution Antimicrobial Suscepibility Tests for Bacteria that Grow Aerobically; Approved Standard Eleven Edition. CLSI Document M07–A11 ISBN 1-56238-784-7. Clinical and Laboratory Standards Institute, 950 West Valley Road, Suite 2500, Wayne, Pennsylvania 19087, USA.

[21] Ferreira TV, Mizuta AG, Menezes JL, de Dutra TV, Bonin E, Castro JC, Abreu Filho BA de. (2020) Effect of ultraviolet treatment (UV–C) combined with nisin on industrialized orange juice in Alicyclobacillus acidoterrestris spores. LWT 133:109911. 10.1016/j.lwt.2020.109911

[22] Eliopoulos GM, Moellering RC Jr (1996) Antimicrobial combinations. In: Lorian V (ed) Antibiotics in Laboratory Medicine. Williams & Wilkins, Baltimore, pp 330–396

[23] Silva DAM, Fernandes MS, Endo EH, Vital ACP, Britta EA, Favero ME, Castro JC, Matumoto-Pintro PT, Dias Filho BP, Nakamura CV, Machinski Junior M, Mikcha JMG, Abreu Filho BA (2021b) Control of the growth of *Alicyclobacillus acidoterrestris* in industrialized orange juice using rosemary essential oil and nisin. Lett Appl Microbiol 72(1):41–49. 10.1111/lam.13385 32910828 10.1111/lam.13385

[24] Vieira TF, Makimori GYF, Santos SMB, Zielinski AAF, Bona E (2020) Chemometric Approach Using ComDim and PLS-DA for Discrimination and Classification of Commercial Yerba Mate (Ilex paraguariensis St. Hil). Food Anal Methods 13:97–107. 10.1007/s12161-019-01520-9

[25] Makimori GYF, Bona E (2019) Commercial instant coffee classification using an electronic nose in tandem with the ComDim-LDA approach. Food Anal Methods 12(5):1067–1076. 10.1007/s12161-019-01443-5

[26] Bona E, Makimori GYF, de Oliveira LFP (2018) Enose CAD. Brazilian patent BR512018001472–0. National Institute of Industrial Property, Link

[27] Galvan D, Bona E (2024) Gamma-gui application: A user-friendly graphic interface for planning experiments in MATLAB. Quim Nova 47(5):1–17. 10.21577/0100-4042.20240005

[28] Thaipong K, Boonprakob U, Crosby K, Cisneros-Zevallos L, Byrne DH (2006) Comparison of antioxidant activity and total phenolic content of different Thai fruit extracts. Food Chem 103(2):381–388. 10.1016/j.foodchem.2006.07.038

[29] Benzie IFF, Strain JJ (1996) The ferric reducing ability of plasma (FRAP) as a measure of antioxidant power: The FRAP assay. Anal Biochem 239(1):70–76. 10.1006/abio.1996.0292 8660627 10.1006/abio.1996.0292

[30] Endo EH, Cortez DAG, Ueda-Nakamura T, Nakamura CV, Dias Filho BP (2010) Potent antifungal activity of extracts and pure compound isolated from pomegranate peels and synergism with fluconazole against *Candida albicans*. Res Microbiol 161(7):534–540. 10.1016/J.RESMIC.2010.05.002 20541606 10.1016/j.resmic.2010.05.002

[31] Li Y, Liu S, Zhao C, Zhang Z, Nie D, Tang W, Li Y (2022) The chemical composition and antibacterial and antioxidant activities of five citrus essential oils. Molecules 27(20):7044. 10.3390/molecules27207044 36296637 10.3390/molecules27207044 PMC9607008

[32] Ruiz SP, Anjos MMD, Carrara VS, Delima JN, Cortez DAG, Nakamura TU, Abreu Filho BA (2013) Evaluation of the antibacterial activity of Piperaceae extracts and nisin on *Alicyclobacillus acidoterrestris*. J Food Sci 78(11):M1772–M1777. 10.1111/1750-3841.12283 24138211 10.1111/1750-3841.12283

[33] Oliveira PRS, Pretes NS, Ribeiro AC, Castro JC, Garcia FP, Nakamura CV, Abreu Filho BA (2024) Comparative assessment of antibacterial activity of *Matricaria chamomilla* L. extract, nisin and of its combination against *Alicyclobacillus* spp. Food Microbiol 124:104597. 10.1016/j.fm.2024.104597 39244376 10.1016/j.fm.2024.104597

[34] Lin H, Li Z, Sun Y, Zhang Y, Wang S, Zhang Q, Cai T, Xiang W, Zeng C, Tang J (2024) D-Limonene: promising and sustainable natural bioactive compound. Appl Sci 14(11):4605. 10.3390/app14114605

[35] He S, Wei Y, Yang Z, Zhang L, Shan A (2025) Synergism between nisin and citronellal against *Fusarium graminearum* and their application in maize preservation. Int J Food Microbiol 111331. 10.1016/j.ijfoodmicro.2025.111331 40609329 10.1016/j.ijfoodmicro.2025.111331

[36] Guo J, Liu Y, Wei L, Wang X, Zhu Y, Yu W, Ma J (2025) Insight into the synergistic antibacterial effect of nisin and Chinese chive seed extract against *Staphylococcus aureus* and their application in pasteurized milk. Food Microbiol 104816. 10.1016/j.fm.2025.104816 40484536 10.1016/j.fm.2025.104816

[37] Bertuso P, Mayer DMD, Nitschke M (2021) Combining celery oleoresin, limonene and rhamnolipid as new strategy to control endospore-forming *Bacillus cereus*. Foods 10(2):455. 10.3390/foods10020455 33669618 10.3390/foods10020455 PMC7922389

[38] Qi H, Chen S, Zhang J, Liang H (2022) Robust stability and antimicrobial activity of d-limonene nanoemulsion by sodium caseinate and high pressure homogenization. J Food Eng 334:111159. 10.1016/j.jfoodeng.2022.111159

[39] Jia H, Zhang W, Wang M, Xie Y, Zhao R, Yue T (2025) A nisin-mediated organic-inorganic hybrid for the inhibition of *Alicyclobacillus acidoterrestris* in apple juice. Food Biosci 107638. 10.1016/j.fbio.2025.107638

[40] Gobbi, E., Falasconi, M., Concina, I., Mantero, G., Bianchi, F., Mattarozzi, M.,… Sberveglieri, G., 2010. Electronic nose and *Alicyclobacillus* spp. spoilage of fruit juices: An emerging diagnostic tool. Food Control 21(10),1374–1382. 10.1016/j.foodcont.2010.04.011

[41] Shah BB, Mehta AA (2018) In vitro evaluation of antioxidant activity of D-limonene. Asian J Pharm Pharmacol 4(6):883–887. 10.31024/ajpp.2018.4.6.25

[42] Al Kamaly O, Numan O, Almrfadi OM, Alanazi AS, Conte R (2022) Separation and evaluation of potential antioxidant, analgesic, and anti-inflammatory activities of limonene-rich essential oils from *Citrus sinensis* (L.). Open Chem 20(1):1517–1530

[43] Berney M, Hammes F, Bosshard F, Weilenmann H-U, Egli T (2007) Assessment and interpretation of bacterial viability by using the LIVE/DEAD BacLight kit in combination with flow cytometry. Appl Environ Microbiol 73(10):3283–3290. 10.1128/AEM.02750-06 17384309 10.1128/AEM.02750-06 PMC1907116

