# Supplementary material for: Association of D-limonene and nisin: the action on vegetative cells and spores of Alicyclobacillus spp. in processed orange juice
Source: Braz J Microbiol. 2026 Jul 24;57(1):216. doi: 10.1007/s42770-026-02024-5 (PMC13396087; doi:10.1007/s42770-026-02024-5)
Supplement: Supplementary file 1 — Supplementary Material 1 (DOCX 16.2 KB) [file 42770_2026_2024_MOESM1_ESM.docx]

**Table S1**, Supplementary Material

Univariate statistical analysis of maximum intensity values for each sensor.

| **Sensors** | **A** | **H** | **I** | **J** |
| --- | --- | --- | --- | --- |
| **MQ3** | 0.3651^a^ ± 0.08 | 0.4521^a^ ± 0.01 | 0.4237^a^ ± 0.00 | 0.4412^a^ ± 0.01 |
| **MQ2** | 0.2889^a^ ± 0.06 | 0.2982^a^ ± 0.00 | 0.2753^a^ ± 0.01 | 0.2841^a^ ± 0.00 |
| **MQ9** | 0.0565^a^ ± 0.01 | 0.0730^a^ ± 0.01 | 0.0762^a^ ± 0.00 | 0.0738^a^ ± 0.00 |
| **MQ138** | 0.0140^a^ ± 0.00 | 0.0134^a^ ± 0.00 | 0.0156^a^ ± 0.00 | 0.0127^a^ ± 0.00 |
| **MQ137** | 0.0197^b^ ± 0.00 | 0.0124^a^ ± 0.00 | 0.0120^a^ ± 0.00 | 0.0109^a^ ± 0.00 |
| **MQ8** | 0.0231^a^ ± 0.00 | 0.0262^a^ ± 0.00 | 0.0314^a^ ± 0.00 | 0.0246^a^ ± 0.00 |

Same letters in the same row indicate no statistically significant difference (p > 0.05) for the mean according to Tukey's test. *Control (A), D-limonene (H), Nisin (I), and combination (J).
